# In Silico Identification of Plant-Derived GPX4 Inhibitors as Potential Ferroptosis Inducers: Molecular Docking, Dynamics, and ADMET Studies

**DOI:** 10.3390/cimb48070668

**Published:** 2026-06-29

**Authors:** Şerife Efsun Antmen, Hasan Öz, Cem Yalaza, Necmiye Canacankatan

**Affiliations:** 1 Department of Biochemistry, Faculty of Pharmacy, Mersin University, 33169 Mersin, Türkiye; 2 Department of Chemistry, Mersin Public Health Laboratory, 28114 Mersin, Türkiye; hasan.oz1@saglik.gov.tr; 3 Department of Biochemistry, Faculty of Pharmacy, Başkent University, 06790 Ankara, Türkiye

**Keywords:** ferroptosis, GPX4, bioavailability, withaferin A, molecular docking

## Abstract

This study aims identify plant-derived compounds that can inhibit glutathione peroxidase 4 (GPX4) enzyme and evaluate them through molecular docking, dynamics simulations, and ADMET analyses. The 3D structure of the GPX4 protein (PDB ID: 2OBI) was obtained from the Protein Data Bank. The plant-derived ligand library was compiled from the PubChem database and screened for compliance with Lipinski’s rules using ADMETLAB 2.0. Molecular docking simulations were performed using Autodock Vina. Molecular dynamics simulations of 100 nanoseconds were performed for the selected ligand–protein complexes using AMBER Tools and OpenMM software. The ADMET properties of the ligands were evaluated using the pKCSM web server. Compared to the reference inhibitor RSL3 (−7.2 kcal/mol), five plant compounds showed stronger binding affinity: withaferin A (−8.0 kcal/mol), mahanine (−7.9 kcal/mol), pseudobufarenogin (−7.8 kcal/mol), cucurbitacin I (−7.6 kcal/mol), and liquiritin (−7.5 kcal/mol). Molecular dynamics simulations showed that the complexes of withaferin A, mahanine, and liquiritin exhibited superior structural stability. ADMET analysis revealed that the compounds generally possess acceptable pharmacokinetic profiles but require some bioavailability optimization. The identified plant-derived compounds can be considered as potential therapeutic agents in cancer treatment by inducing ferroptosis via GPX4 inhibition. These findings provide an important basis for natural product-derived drug discovery studies.

## 1. Introduction

In recent years, the role of programmed cell death mechanisms in cancer treatment has attracted attention. Ferroptosis is a newly discovered form of cell death that differs from traditional apoptosis and necrosis, arising from iron-dependent lipid peroxide accumulation [1,2]. Ferroptosis is fundamentally based on the disruption of the redox imbalance between oxidants and antioxidants [3]. In this process, iron ions catalyze the oxidation of lipids, leading to high levels of lipid peroxidation within the cell [4]. This triggers a significant cellular stress response that leads to disruption of cell membrane integrity and ultimately cell death [5]. This iron-dependent lipid peroxidation process is characterized by the oxidation of phospholipids containing polyunsaturated fatty acids in cell membranes, distinguishing it from other cell death mechanisms [6]. This mechanism is closely related to the decrease in glutathione peroxidase 4 activity. Glutathione peroxidase 4 (GPX4) is the main regulator of cellular protection against ferroptosis. This enzyme reduces phospholipid hydroperoxides to harmless lipid alcohols, thereby preventing ferroptosis [7]. Loss of GPX4 activity triggers this iron-dependent cell death, particularly by leading to the accumulation of lipid-based reactive oxygen species and lipid hydroperoxides [8]. Therefore, the induction and inhibition of ferroptosis are attracting attention as a novel therapeutic approach for cancer treatment and neurodegenerative diseases [9]. Consequently, GPX4 targeted therapeutic approaches offer an innovative strategy for tumors resistant to conventional cancer treatments [10].

Among existing GPX4 inhibitors, RSL3 (RAS-selective lethal 3) is one of the best characterized compounds. RSL3 irreversibly inhibits the enzyme by covalently binding to the selenocysteine residue in the active site of GPX4 [11]. However, the potential toxicity profiles of synthetic inhibitors complicate their clinical application. At this point, natural products derived from plant sources emerge as valuable resources in drug discovery. These compounds generally exhibit better tolerance profiles compared to their synthetic analogues [12].

The present study constitutes an in silico investigation to evaluate the binding affinities of five distinct plant-derived compounds withaferin A, mahanine, pseudobufarenogin, cucurbitacin I, and liquiritin against the active site of the GPX4 enzyme, using RSL3 as a reference inhibitor. These five compounds were selected based on their documented anti-cancer activity in the literature, structural diversity (spanning steroidal lactone, carbazole alkaloid, cardiac glycoside, triterpenoid, and flavonoid glycoside scaffolds), compliance with Lipinski’s rule-of-five, and availability of high-quality 3D structures in PubChem. It should be noted that some of the compounds studied have previously been investigated in the context of ferroptosis, and the present study complements those findings by providing direct binding evidence at the GPX4 active site through a comparative computational framework. Using molecular docking, molecular dynamics simulation analyses, and ADMET profile evaluations, the goal is to identify natural product derivatives with potential as ferroptosis-inducing agents in cancer therapy. This comprehensive approach will contribute to the development of innovative therapeutic strategies in the field of GPX4-targeted cancer therapy.

## 2. Methods

This study was performed using Autodock Vina [13] molecular docking simulation. The molecular docking simulation results were visualized using Discovery Studio Visualizer [14]. Molecular dynamics simulations of ligand–protein complexes were performed using openMM and amber tools. The ADMET properties of the ligands were examined using the pKCSM web server [15]. The 3D structure of GPX4 was obtained from the Protein Data Bank (https://www.rcsb.org/). The SMILES structures of the ligands were obtained from PubChem (https://www.ncbi.nlm.nih.gov/) and converted to pdb format using the NovoPro online tool (https://www.novoprolabs.com/tools/smiles2pdb, accessed on 20 May 2026).

### 2.1. Optimization of the GPX4 Protein

The 3D structure of the GPX4 protein was obtained from the protein database with PDB ID: 2OBI. Autodock Tools was used to optimize the structure of GPX4. This optimization was performed to remove water and unwanted molecules bound to the protein. Polar hydrogen bonds were added, and Gasteigner and Kollman charges were calculated.

### 2.2. Creating a Ligand Library

Plant-derived GPX4 inhibitor candidate ligands were obtained from the PubChem database. The ligand library consists of 261 compounds. These compounds were selected based on: (1) documented anti-cancer activity across diverse mechanisms in the peer-reviewed literature; (2) structural diversity to probe multiple binding modes within the GPX4 active site; (3) compliance with Lipinski’s [16] rule-of-five as screened by ADMETlab 2.0 [17]; and (4) availability of high-quality 3D SMILES structures in PubChem. This represents a targeted, hypothesis-driven selection informed by prior biological activity data rather than a systematic database screen. Ligands that complied with Lipinski’s rules were optimized using Autodock Tools.

### 2.3. Molecular Docking Simulations

The selected ligands prepared in the previous step were docked to GPX4 twice using the Autodock Vina software. The binding site was determined using the CB-Dock2 web server. For this purpose, the standard molecule for the GPX4 receptor, RSL3 (methyl (1S,3R)-2-(2-chloroacetyl)-1-(4-methoxycarbonylphenyl)-1,3,4,9-tetrahydropyrido [3,4-b]indole-3-carboxylate) was subjected to blind molecular docking simulation via CB-Dock2, and the binding site was identified. The binding site coordinates for the molecular docking simulation were set to x = 32.0012, y = −28.0256, z = −9.0403, and the grid size was set to x = 20, y = 20, z = 20. Autodock Vina was used for the molecular docking of the ligands to the target protein. The structure with the lowest binding energy for each ligand was determined to be the best structure. After the docking process, the interactions between the receptor and ligand molecules were examined and visualized using the Discovery Studio Visualizer program.

### 2.4. Molecular Dynamics (MD) Simulations

To investigate the interaction dynamics of the GPX4 protein with inhibitor ligands, AMBER Tools [18] and OpenMM [19] software were used to investigate the interaction dynamics of the GPX4 protein with inhibitor ligands. Crystallographic water molecules were removed from the protein structure, and hydrogen atoms were added to the ligands. The charge states of the ligands were optimized considering physiological pH (7.4). In molecular modeling, the ff19SB [20] model was used for the protein, the TIP3P model [21], and the GAFF2 [22] force field for the ligands. The system was equilibrated at 298 K and 1 bar pressure. A free MD simulation was performed for 100 ns.

### 2.5. Review of ADMET Properties

The ADMET properties of ligands with the best docking scores were examined using the pKCSM (https://biosig.lab.uq.edu.au/pkcsm/, accessed on 20 May 2026) web server. The SMILES structures of the ligands with the best docking scores were loaded into pKCSM to obtain their ADMET properties.

## 3. Result

### 3.1. Molecular Docking Simulation

**Figure 1:**
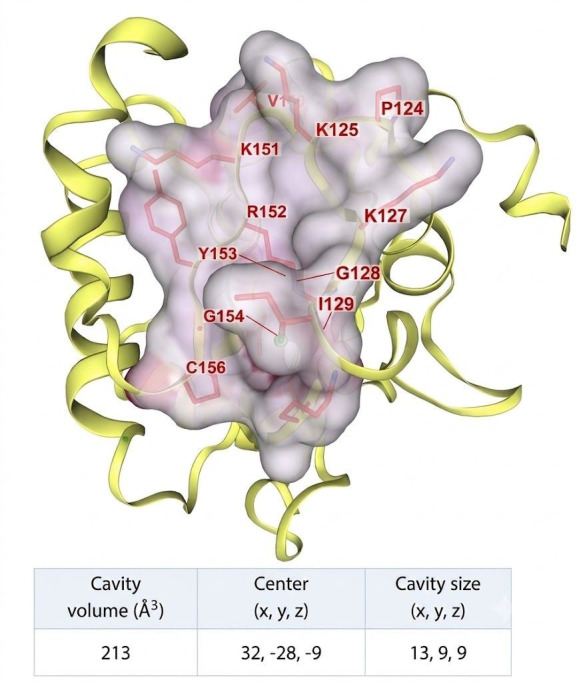
GPX4 (PDB ID: 2OBI) 3D structure. The active site region used for molecular docking is indicated.

Glutathione peroxidase 4 is an enzyme considered a potential candidate in cancer treatment. The study utilized the 3D structure of GPX4 from the protein data bank PDB ID: 2OBI. This structure was obtained at a resolution of 1.55 Å using the X-ray diffraction method [11]. The relevant structure is shown in Figure 1, with the active site pocket (identified by CB-Dock2) provide structural context for the docking results.

Docking results for plant compounds that inhibit the GPX4 enzyme using Autodock Vina, with RSL3 as the reference molecule, are presented in Table 1.

### 3.2. Molecular Dynamics Simulation

**Figure 2:**
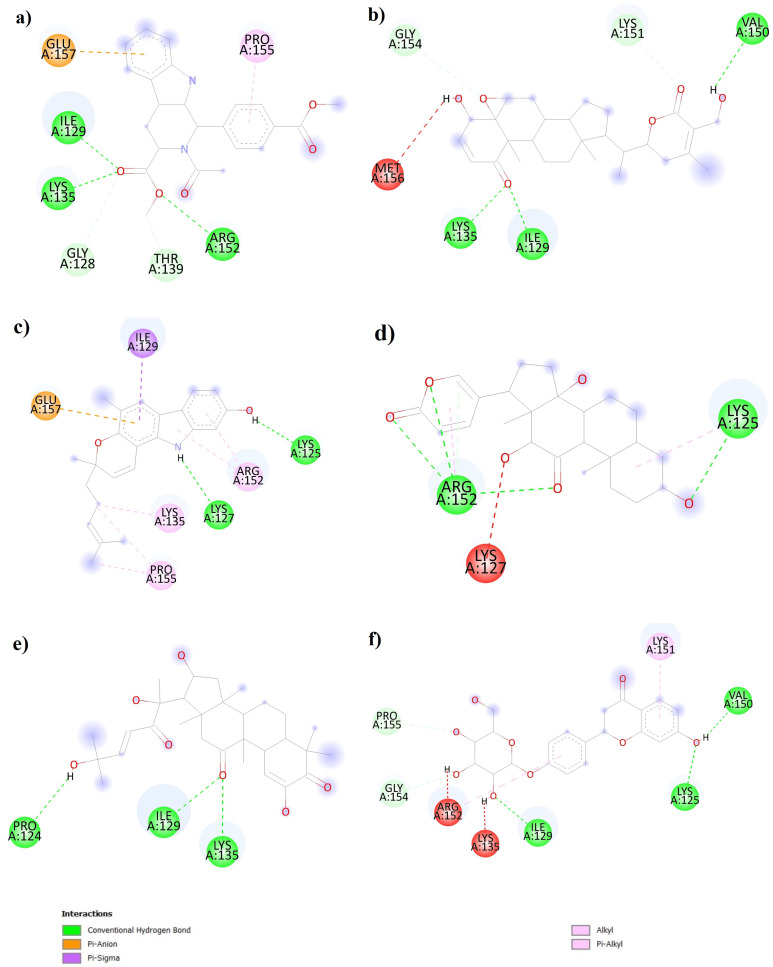
Amino acid interactions between ligands and receptors. (**a**) RSL3-GPX4 interaction. (**b**) Withaferin A–GPX4 interaction. (**c**) Mahanine–GPX4 interaction. (**d**) Pseudobufarenogin–GPX4 interaction. (**e**) Cucurbitacin I–GPX4 interaction. (**f**) Liquiritin–GPX4 interaction.

In this study, the structural stability and conformational dynamics of six different ligand–protein complexes were evaluated using 100 nanosecond molecular dynamics simulations. The binding properties of the RSL3, withaferin A, mahanine, pseudobugarenogin, cucurbitacin I, and liquiritin complexes were comprehensively investigated using RMSD, RMSF, radius of gyration, and principal component analysis methods (Figure 2).

#### 3.2.1. Complex Stability Profiles

**Figure 3:**
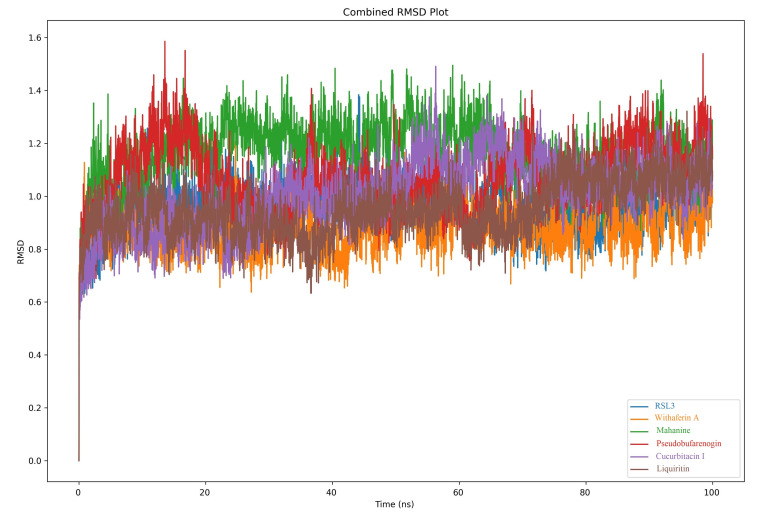
Combined RMSD Graph. Time-dependent change in RMSD values of backbone atoms for six different ligand–protein complexes throughout a 100 ns MD simulation.

RMSD analyses revealed significant differences in stability among the ligands (Figure 3). RMSD values reported reflect the plateau phase of the simulation (ns 20–100). The RSL3, withaferin A, and liquiritin complexes exhibited superior binding stability with low RMSD fluctuations (0.7–1.0 Å). These findings indicate that the ligands in question form strong interactions with the protein and cause minimal structural perturbation. Cucurbitacin I and pseudobugarenogin complexes showed moderate stability in the 0.9–1.2 Å range, while the mahanine complex recorded the highest RMSD values (1.1–1.4 Å). This result indicates that the complex exhibits increased conformational dynamic behavior following mahanine binding.

#### 3.2.2. Residual Level Flexibility Analysis

**Figure 4:**
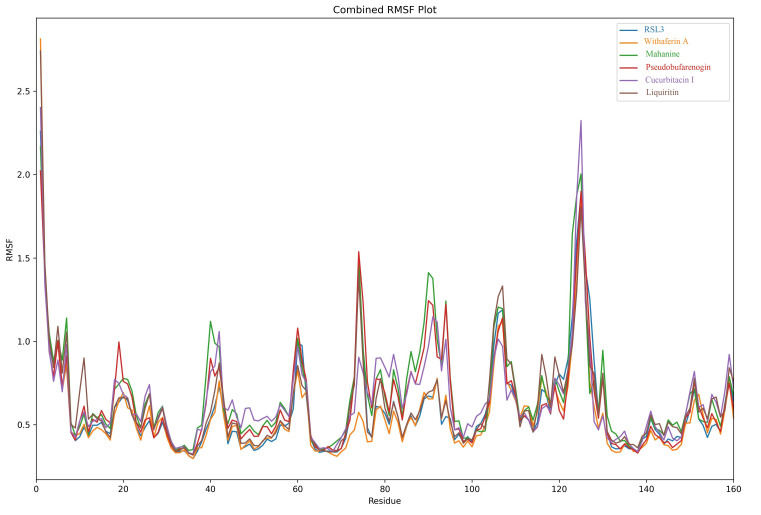
Combined RMSF Plot. RMSF values for each residue in six different ligand–protein complexes throughout a 100 ns MD simulation.

RMSF results indicate that ligand binding preserves the overall folding architecture of the protein but causes localized flexibility changes in specific regions (Figure 4). Similar RMSF profiles observed across all systems reveal that structural modifications are confined to specific regions without affecting global protein stability. These findings support the notion that compounds such as RSL3, withaferin A, and liquiritin contribute to complex stability by providing localized stabilization in flexible regions of the protein.

#### 3.2.3. Protein Compactness Analysis

**Figure 5:**
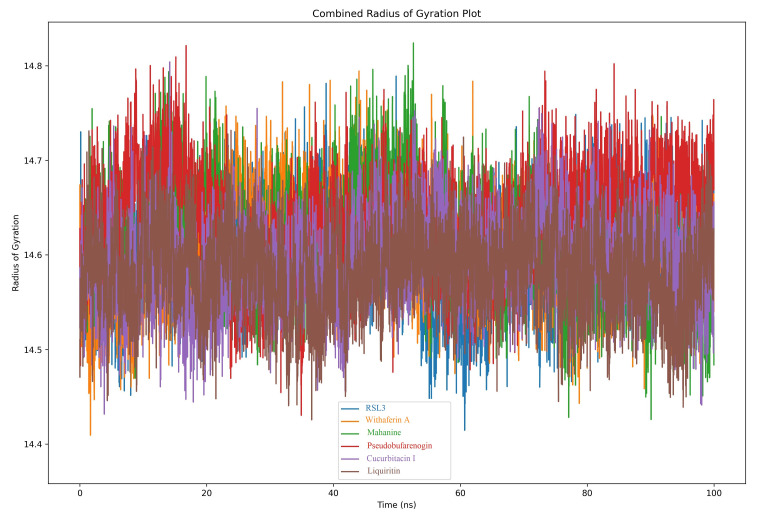
Combined Radius of Gyration Graph. Rg values for each residue in six different ligand–protein complexes throughout a 100 ns MD simulation.

Turning radius measurements showed that all complexes maintained stable Rg values throughout the simulation (Figure 5). These results confirm that ligand binding did not cause significant protein unfolding or denaturation events and demonstrate that overall structural integrity was preserved.

#### 3.2.4. Conformational Dynamic Characterization

**Figure 6:**
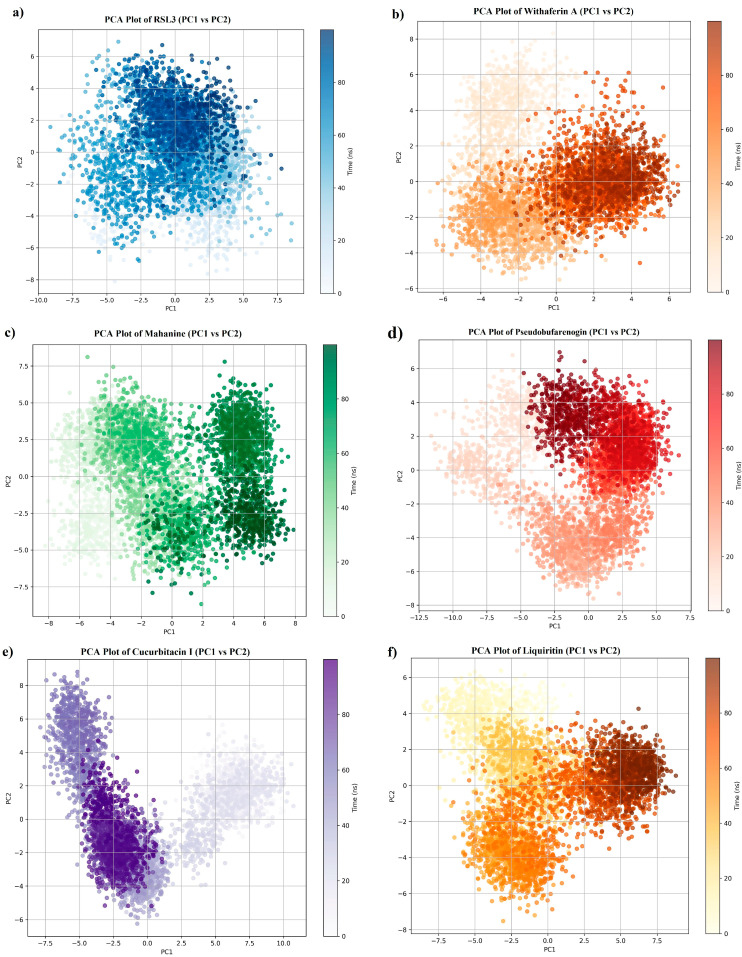
PCA Plots (PC1 vs PC2) with time progression (100 ns Simulation). (**a**) PCA Plot of RSL3. (**b**) PCA Plot of Withaferin A. (**c**) PCA Plot of Mahanine. (**d**) PCA Plot of Pseudobufarenogin (**e**) PCA Plot of Cucurbitacin I. (**f**) PCA Plot of Liquiritin.

Principal component analysis revealed distinct conformational sampling behavior differences among ligands (Figure 6). It is important to note that RMSD and PCA capture complementary but distinct aspects of protein dynamics. RMSD measures temporal backbone deviation from the reference structure, whereas PCA characterises the dimensionality and distribution of sampled conformational space. Mahanine exhibited movement within the most restricted conformational space (tight clustering around the origin) and displayed minimal spread of sampled conformations. This finding reflects that mahanine’s conformational fluctuations are repetitive and cyclic around a limited set of states. While this appears paradoxical given mahanine’s high RMSD values, the combination indicates that the complex undergoes repeated, structurally significant deviations from the initial reference conformation without exploring new areas of conformational space, consistent with a partially destabilised but conformationally trapped binding mode. Withaferin A showed moderate conformational stability with a central distribution pattern. Cucurbitacin I exhibited the most dramatic conformational transition with a directed migration from dispersed initial conformations to a dense region and showed signs of a potential induced-fit mechanism. RSL3 and liquiritin showed significant structural adaptability with extensive conformational sampling behavior across large regions of PCA space. Pseudobugarenogin exhibited an intermediate position between stable and dynamic behaviors.

### 3.3. Review of ADMET Properties

The pKCSM web server was used to predict the ADMET properties of ligands. The absorption, distribution, metabolism, excretion, and toxicity properties of the compounds with the best docking scores were determined. The results obtained from the pKCSM web server are presented in Table 2.

When Caco-2 permeability is <0.90 and intestinal absorption values are <30%, intestinal absorption will be poor [23]. Withaferin A, cucurbitacin I, and liquiritin have low Caco-2 permeability. The intestinal absorption values of withaferin A, mahanine, pseudobufarenogin, and cucurbitacin I are adequate (>80%). However, liquiritin’s intestinal absorption value of 46.1% falls below the conventional ≥70% threshold for good oral absorption and, combined with its low Caco-2 permeability (0.507), represents a significant pharmacokinetic liability. This dual limitation suggests that liquiritin, despite its favorable GPX4 binding affinity and MD stability, would require substantial formulation optimization (e.g., solid dispersion or nanoparticle encapsulation) or structural modification to achieve adequate oral bioavailability. At this point, bioavailability issues can be resolved by applying micro- and nanocapsulation technologies or other techniques. The permeability of the blood–brain barrier (BBB) is a critical factor in the transport of therapeutic agents to the central nervous system (CNS). Compounds with logBB > 0.3 can cross the BBB, while those with logBB < −1 are poorly distributed [15]. Among the compounds studied, liquiritin has low BBB permeability. Compounds with logPS > −2 can penetrate the central nervous system, while those with logPS < −3 cannot [15]. Accordingly, curcurbitacin I and liquiritin cannot penetrate the central nervous system. P450 inhibitors have the ability to significantly alter the pharmacokinetics of these drugs. CYP2D6 and CYP3A4 are enzymes found in the human liver and play an important role in the metabolism of many drugs and substances. CYP2D6 metabolizes approximately 25% of drugs [24]. None of the compounds studied are metabolized by the CYP2D6 enzyme. Pseudobufarenogin and liquiritin are not substrates of the CYP3A4 enzyme. The total clearance value represents a drug’s capacity to be eliminated by different organs, such as the liver and kidneys. The total clearance value was determined to be highest for the compound pseudobufarenogin. Ames toxicity measures a compound’s ability to be mutagenic [25]. According to the toxicity test (Ames test), mahanine and liquiritin were found to be potentially mutagenic. None of the compounds examined showed hepatotoxic effects.

## 4. Discussion

Developing new formulation strategies to overcome low bioavailability issues may be an important focus for future research. More research is needed on the pharmacokinetic properties of these compounds, their effects on metabolic pathways, and their toxicity profile. These studies are important for fully understanding the therapeutic potential of these compounds and optimizing their use in clinical practice. These plant-derived compounds may offer promising innovative treatment strategies for cancer therapy by inducing ferroptosis through targeting GPX4. This study investigated plant-derived inhibitors of GPX4 and their ADMET properties. Molecular docking simulations identified five plant-derived compounds that could outperform RSL3, the standard inhibitor for GPX4. These are withaferin A, mahanine, pseudobufarenogin, cucurbitacin I, and liquiritin. The interaction of the six identified inhibitor ligands with the protein was found to exhibit distinct differences in their molecular dynamics. RMSD analysis results showed that RSL3, withaferin A, and liquiritin formed the most stable complexes with the protein. The low RMSD values of these ligands indicate strong protein–ligand interactions. The high RMSD values of mahanine, on the other hand, indicate significant conformational changes after binding. RMSF results showed that all ligands preserved the protein folding structure but caused localized flexibility changes in specific regions. This suggests that ligand binding may exert regulatory effects without disrupting protein function. The stability of the radius of gyration values confirms that protein compactness is maintained in the presence of ligands. Withaferin A is a C28 steroidal lactone isolated from the plant *Withania somnifera* (Ashwagandha). Withaferin A has been reported to be effective against many types of cancer, including breast, lung, colon, brain, and cervical cancer [26]. Withaferin A exerts these effects through pro-apoptotic activities by interacting with NF-κB, STAT, Hsp90, ER-α, p53, and TGF-β [27]. Mahanine is a carbazole alkaloid isolated from the leaves of *Murraya koenigii*. In breast cancer, mahanine has been shown to have a potent inhibitory effect on estrogen receptor-positive (ER+) breast tumors [28]. Mahanine exerts its anti-cancer effects through the blockade of STAT3, RASSF1A, and AKT/mTOR pathways, and the inactivation of the Hsp90-cdc37 complex and mitochondrial complex III [29]. Pseudobufarenogin is a cardiac glycoside belonging to the bufadienolide class and exerts its effects by inhibiting the Na^+^/K^+^-ATPase enzyme. Pseudobufarenogin inhibits cell proliferation in hepatocellular carcinoma. It also exhibits anti-cancer effects by disrupting the MEK/ERK and PI3K/Akt pathways [30]. Cucurbitacin I is a secondary metabolite with a tetracyclic triterpenoid structure found in plants belonging to the Cucurbitaceae family [31]. At the molecular level, the anti-cancer effects of cucurbitacin I occur through various mechanisms, such as inhibition of the JAK2/STAT3 pathway and activation of apoptotic pathways [32]. Liquiritin is a flavonoid glycoside obtained from the roots or rhizomes of the plants *Glycyrrhiza uralensis* Fisch., *Glycyrrhiza inflata* Bat., and *Glycyrrhiza glabra* L. (licorice root) [33]. Liquiritin has shown significant potential in cancer treatment: It exhibits potent inhibitory effects on various cancer cell types, including breast, lung, liver, and colon cancers, by modulating various signaling pathways such as PI3K/AKT/mTOR, MAPK/ERK, JAK/STAT, and Wnt/β-catenin [33].

The molecular dynamics simulations provided a critical validation step, confirming that the identified ligand–protein complexes maintain their structural integrity and binding posture under physiological conditions. This sustained stability is particularly significant as it suggests a prolonged drug-target residence time, a parameter often more predictive of in vivo efficacy than static binding affinity alone. Regarding the ADMET profile, although some candidates exhibited limitations such as low CNS permeability or metabolic susceptibility, these are common characteristics of natural product scaffolds. From a drug discovery perspective, these compounds should be viewed as promising ‘lead structures’ rather than final drugs. Their pharmacokinetic limitations can be effectively overcome through medicinal chemistry strategies, such as semisynthetic derivatization to optimize lipophilicity, or by employing advanced drug delivery systems like nanoparticle encapsulation, thereby preserving their potent GPX4 inhibitory activity while enhancing bioavailability.

An important mechanistic consideration concerns the choice of RSL3 as a reference inhibitor. RSL3 functions as an irreversible, covalent inhibitor of GPX4, reacting with the catalytic selenocysteine residue via its chloroacetyl moiety. The plant-derived candidates evaluated in this study are non-covalent (reversible) binders. As such, the molecular docking binding affinity scores reported here represent non-covalent interaction free energies and are not directly comparable to the pharmacological IC50 of RSL3. RSL3 was employed in this study as a geometric reference to validate the binding site coordinates (identified by CB-Dock2) and to provide a baseline for non-covalent interaction profiling within the same binding pocket. Researchers interested in quantitative potency comparisons should refer to covalent docking approaches or experimental enzymatic assays.

Regarding mutagenicity and CNS penetration, two important safety and pharmacological concerns merit dedicated discussion. Mahanine and liquiritin were identified as Ames test-positive, indicating mutagenic potential that constitutes a significant obstacle for direct clinical translation. Future medicinal chemistry efforts should focus on identifying the structural motifs responsible for mutagenicity and designing semi-synthetic analogues from which these groups are removed while preserving GPX4 binding affinity. The poor BBB and CNS penetration of cucurbitacin I (logBB = −0.915; logPS = −3.43) and liquiritin (logBB = −1.146; logPS = −3.866) limits their use for CNS malignancies but may be viewed as an advantage for peripheral tumor targeting, where off-target CNS effects would be minimized.

Strengths of this study include: (1) the use of a high-resolution, experimentally validated GPX4 crystal structure (PDB: 2OBI, 1.55 Å resolution); (2) a comprehensive, multi-metric MD analysis integrating RMSD, RMSF, Rg, and PCA to provide a robust assessment of binding stability; and (3) integrated ADMET profiling within a single computational framework enabling direct inter-compound comparison. Limitations include: (1) the absence of wet-lab experimental validation (enzymatic inhibition assays, cell-based ferroptosis induction assays); (2) the use of a single GPX4 crystal structure, which does not account for conformational flexibility of the apo protein; (3) Ames mutagenicity signals for mahanine and liquiritin, which require structural optimization before clinical consideration; and (4) the non-covalent docking framework, which does not fully model RSL3’s covalent binding mechanism. Future studies should incorporate experimental validation, ensemble docking approaches, and covalent docking methodologies where appropriate.

## 5. Conclusions

This study provides comprehensive biochemical evidence for the targeting of the GPX4 enzyme, the main regulator of the ferroptosis mechanism, with plant-derived compounds. The findings are derived from in silico analyses and represent lead identification results that require experimental validation before clinical implications can be drawn. The results demonstrate that naturally derived small molecules offer structural diversity and binding stability that could overcome the pharmacokinetic limitations of existing synthetic inhibitors.

The key outcome of our study is that compounds such as withaferin A and liquiritin not only bind with high affinity but also possess a sustainable inhibition potential by preserving enzyme integrity under physiological dynamics. Although the identified mutagenicity risks pose an obstacle for clinical application, the identification of these compounds as ‘lead structures’ provides a strategic foundation for future semi-synthetic drug design studies. It is important to acknowledge that RSL3, used as a reference, is a covalent irreversible inhibitor, and the non-covalent binding scores reported for the plant-derived candidates are therefore not directly equivalent measures of inhibitory potency. In conclusion, this study combines the disciplines of biochemistry and natural products chemistry around the axis of ferroptosis, providing a priority roadmap for developing new-generation therapeutic approaches against chemoresistant cancer types.

## Figures and Tables

**Table 1:** Binding affinities (kcal/mol) and interactions of inhibitor ligands with GPX4 in molecular docking simulations.

	Compounds	Binding Affinity (kcal/mol)	Interactions
0	RSL3	−7.2	GLY128, **ILE129**, **LYS135**, THR139, **ARG152**, PRO155, GLU157
1	Withaferin A	−8.0	**ILE129**, **LYS135**, **VAL150**, LYS151, GLY154, MET156
2	Mahanine	−7.9	**LYS125**, **LYS127**, ILE129, LYS135, ARG152, PRO155, GLU157
3	Pseudobufarenogin	−7.8	**LYS125**, LYS127, **ARG152**
4	Cucurbitacin I	−7.6	**PRO124**, **ILE129**, **LYS135**
5	Liquiritin	−7.5	**LYS125**, **ILE129**, LYS135, **VAL150**, LYS151, ARG152, GLY154, PRO155

Ligands form hydrogen bonds with the bolded amino acids of GPX4.

**Table 2:** ADMET properties of GPX4 inhibitors.

ADMET	Properties	Withaferin A	Mahanine	Pseudobufarenogin	Cucurbitacin I	Liquiritin
Absorption	Caco-2	0.829	1.185	1.164	0.649	0.507
	İnt. Abs	85.345	88.259	86.473	85.113	46.076
Distribution	BBB	−0.03	−0.036	−0.553	−0.915	−1.146
	CNS	−2.72	−1.921	−2.376	−3.43	−3.866
Metabolism	CYP2D6	No	No	No	No	No
	CYP3A4	Yes	Yes	No	Yes	No
Excretion	TC	0.435	0.249	0.469	0.188	0.342
Toxicity	Ames	No	Yes	No	No	Yes
	Htox	No	No	No	No	No

**Abbreviations:** Caco-2 permeability (log Papp 10–6 cm/s); Int. Abs: Intestinal absorption (% Absorbed); BBB permeability: Blood–brain barrier permeability (log BB); CNS permeability: Central nervous system permeability (log PS); TC: Total clearance (log ml/min/kg); Ames: Measures the compound’s mutagenic potential; Htox: Hepatotoxicity.

## Data Availability

The original contributions presented in this study are included in the article. Further inquiries can be directed to the corresponding author.

## References

[1] Chen H., Wang C., Liu Z., He X., Tang W., He L., Wang Y., Li T. (2022). Ferroptosis and its multifaceted role in cancer: Mechanisms and therapeutic approach. Antioxidants.

[2] Luo Y., Bai X.Y., Zhang L., Hu Q.Q., Zhang N., Cheng J.Z., Liu X.L. (2024). Ferroptosis in cancer therapy: Mechanisms, small molecule inducers, and novel approaches. Drug Des. Dev. Ther..

[3] Lan H., Gao Y., Zhao Z., Mei Z., Wang F. (2022). Ferroptosis: Redox imbalance and hematological tumorigenesis. Front. Oncol..

[4] Tan S., Kong Y., Xian Y., Gao P., Xu Y., Wei C., Zhu X. (2022). The mechanisms of ferroptosis and the applications in tumor treatment: Enemies or friends?. Front. Mol. Biosci..

[5] Zheng X., Jin X., Ye F., Liu X., Yu B., Li Z., Li Q. (2023). Ferroptosis: A novel regulated cell death participating in cellular stress response, radiotherapy, and immunotherapy. Exp. Hematol. Oncol..

[6] Li X., Meng F., Wang H., Sun L., Chang S., Li G., Chen F. (2024). Iron accumulation and lipid peroxidation: Implication of ferroptosis in hepatocellular carcinoma. Front. Endocrinol..

[7] Oh S.W., Harris J.A., Ng L., Winslow B., Cain N., Mihalas S., Hui Q., Kuan C.A., Henry A.M., Mortrud M.T. (2014). A mesoscale connectome of the mouse brain. Nature.

[8] Yang W.S., Stockwell B.R. (2016). Ferroptosis: Death by lipid peroxidation. Trends Cell Biol..

[9] Saimoto Y., Kusakabe D., Morimoto K., Matsuoka Y., Kozakura E., Kato N., Yamada K.I. (2025). Lysosomal lipid peroxidation contributes to ferroptosis induction via lysosomal membrane permeabilization. Nat. Commun..

[10] Iglesias-Matesanz P., Lacalle-Gonzalez C., Lopez-Blazquez C., Ochieng’Otieno M., Garcia-Foncillas J., Martinez-Useros J. (2024). Glutathione peroxidases: An emerging and promising therapeutic target for pancreatic cancer treatment. Antioxidants.

[11] Scheerer P., Borchert A., Krauss N., Wessner H., Gerth C., Höhne W., Kuhn H. (2007). Structural basis for catalytic activity and enzyme polymerization of phospholipid hydroperoxide glutathione peroxidase-4 (GPx4). Biochemistry.

[12] Koehn F.E., Carter G.T. (2005). The evolving role of natural products in drug discovery. Nat. Rev. Drug Discov..

[13] Morris G.M., Huey R., Lindstrom W., Sanner M.F., Belew R.K., Goodsell D.S., Olson A.J. (2009). AutoDock4 and AutoDockTools4: Automated docking with selective receptor flexibility. J. Comput. Chem..

[14] BIOVIA Discovery Studio (2017). Dassault Systemes, Discovery Studio.

[15] Pires D.E., Blundell T.L., Ascher D.B. (2015). pkCSM: Predicting small-molecule pharmacokinetic and toxicity properties using graph-based signatures. J. Med. Chem..

[16] Lipinski C.A., Lombardo F., Dominy B.W., Feeney P.J. (2012). Experimental and computational approaches to estimate solubility and permeability in drug discovery and development settings. Adv. Drug Deliv. Rev..

[17] Xiong G., Wu Z., Yi J., Fu L., Yang Z., Hsieh C., Cao D. (2021). ADMETlab 2.0: An integrated online platform for accurate and comprehensive predictions of ADMET properties. Nucleic Acids Res..

[18] Case D.A., Aktulga H.M., Belfon K., Cerutti D.S., Cisneros G.A., Cruzeiro V.W.D., Forli S., Giotto S., Gohlke H., Götz A.W. (2023). AmberTools. J. Chem. Inf. Model..

[19] Eastman P., Galvelis R., Peláez R.P., Abreu C.R., Farr S.E., Gallicchio E., Ganesan A., Gapsys V., Meyer R.K., Pirritano M. (2023). OpenMM 8: Molecular dynamics simulation with machine learning potentials. J. Phys. Chem. B.

[20] Tian C., Kasavajhala K., Belfon K.A., Raguette L., Huang H., Migues A.N., Maezato J., Brozell S.R., Case D.A., Walker R.C. (2019). ff19SB: Amino-acid-specific protein backbone parameters trained against quantum mechanics energy surfaces in solution. J. Chem. Theory Comput..

[21] Boonstra S., Onck P.R., van der Giessen E. (2016). CHARMM TIP3P water model suppresses peptide folding by solvating the unfolded state. J. Phys. Chem. B.

[22] He X., Man V.H., Yang W., Lee T.S., Wang J. (2020). A fast and high-quality charge model for the next generation general AMBER force field. J. Chem. Phys..

[23] Chan R., De Bruyn T., Wright M., Broccatelli F. (2018). Comparing mechanistic and preclinical predictions of volume of distribution on a large set of drugs. Pharm. Res..

[24] Wang B., Yang L.P., Zhang X.Z., Huang S.Q., Bartlam M., Zhou S.F. (2009). New insights into the structural characteristics and functional relevance of the human cytochrome P450 2D6 enzyme. Drug Metab. Rev..

[25] Dutta R., Khalil R., Green R., Mohapatra S.S., Mohapatra S. (2019). Withania somnifera (Ashwagandha) and withaferin A: Potential in integrative oncology. Int. J. Mol. Sci..

[26] Atteeq M. (2022). Evaluating anticancer properties of Withaferin A—A potent phytochemical. Front. Pharmacol..

[27] Samanta S.K., Choudhury P., Kandimalla R., Aqil F., Moholkar D.N., Gupta R.C., Talukdar N.C. (2024). Mahanine mediated therapeutic inhibition of estrogen receptor-α and CDK4/6 expression, decipher the chemoprevention-signaling cascade in preclinical model of breast cancer. J. Ethnopharmacol..

[28] Samanta S.K., Kandimalla R., Gogoi B., Dutta K.N., Choudhury P., Deb P.K., Devi R., Pal C.B., Talukdar N.C. (2018). Phytochemical portfolio and anticancer activity of Murraya koenigii and its primary active component, mahanine. Pharmacol. Res..

[29] Ding J., Wen W., Xiang D., Yin P., Liu Y., Liu C., Wang H. (2015). ψ-Bufarenogin, a novel anti-tumor compound, suppresses liver cancer growth by inhibiting receptor tyrosine kinase-mediated signaling. Oncotarget.

[30] Kapoor N., Ghorai S.M., Kushwaha P.K., Shukla R., Aggarwal C., Bandichhor R. (2020). Plausible mechanisms explaining the role of cucurbitacins as potential therapeutic drugs against coronavirus 2019. Inform. Med. Unlocked.

[31] Zieniuk B., Pawełkowicz M. (2023). Recent advances in the application of cucurbitacins as anticancer agents. Metabolites.

[32] Qin J., Chen J., Peng F., Sun C., Lei Y., Chen G., Xie X. (2022). Pharmacological activities and pharmacokinetics of liquiritin: A review. J. Ethnopharmacol..

[33] Bhat A.A., Moglad E., Afzal M., Agrawal N., Thapa R., Almalki W.H., Gupta G. (2025). The anticancer journey of liquiritin: Insights into its mechanisms and therapeutic prospects. Curr. Med. Chem..

